# Determinants of treatment response to cognitive behavioral therapy in veterans presenting with comorbid insomnia and sleep apnea

**DOI:** 10.1007/s11325-025-03298-z

**Published:** 2025-03-14

**Authors:** Ali A. El-Solh, Amber Martinson, Parveen Attai, Gregory Homish, Keziah Aibangbee, Erin Gould

**Affiliations:** 1 Sleep Disorders Research Center, VA Western New York Healthcare System, Buffalo, NY USA; 2 Department of Medicine, Jacobs School of Medicine, Buffalo, NY USA; 3 Department of Epidemiology and Environmental Health, School of Public Health and Health Professions, Buffalo, NY USA; 4 Behavioral Health Service, George Wahlen VA Medical Center, Salt Lake City, UT USA; 5 Department of Community Health, School of Public Health and Health Professions, University at Buffalo, Buffalo, NY USA; 6 VA Western New York Healthcare System, 3495 Bailey Avenue, Buffalo, NY 14215 USA

**Keywords:** Cognitive behavioral therapy, Insomnia, Sleep apnea, Pharmacotherapy, Continuous positive airway pressure

## Abstract

**Purpose:**

Although cognitive behavioral therapy for insomnia (CBT-I) is considered the preferred treatment for insomnia in patients with comorbid insomnia and obstructive sleep apnea (COMISA), the remission rate with CBT-I is generally considered lower than in insomnia-only populations. There is also a sizable variability in individual treatment responses. Due to the limited availability of CBT-I, we sought to identify specific clinical attributes that predict benefit from Brief Behavioral Therapy for Insomnia (BBTI)-an adaptation of CBT-I-in patients with COMISA.

**Methods:**

We conducted a retrospective analysis of the National Veterans Health Administration (VHA) electronic medical records covering veterans diagnosed with COMISA between January 2021 and December 2023. Insomnia Severity Index (ISI) scores were recorded at baseline and after 12±1 weeks after BBTI. A positive response to BBTI was defined as a reduction in ISI score of ≥ 8 from baseline. A multivariate generalized linear model analysis was performed to delineate predictive factors of BBTI responsiveness.

**Results:**

131 eligible cases received BBTI over 6 weeks, 56 (43%) of whom did not respond. Non-whites (OR 3.5, 95% CI [1.4, 8.8]) and shorter sleep time (OR 0.98, 95% CI [0.98, 0.99] were independent predictors of blunted response to BBTI. These findings remained true even when depression and AHI were forced into the regression model. Patients with a total sleep duration of < 4.1 h were at greatest risk of being nonresponsive to BBTI.

**Conclusion:**

These findings indicate that identifying insomnia phenotypes in patients with COMISA would help deliver personalized care while maximizing BBTI treatment resources.

## Introduction

Insomnia and obstructive sleep apnea (OSA) are the two most prevalent sleep disorders in the general population [1, 2]. Although both conditions have different underlying pathophysiology, it is not uncommon for these two entities to coexist. The first description of the “new syndrome” - dubbed as COMISA-was published in 1973 by Guilleminault, Eldridge, and Demont [3], however, it was not until 1999 that the significance of the co-occurrence of these disorders on overall health was recognized [4]. An accurate estimate of the prevalence of COMISA is lacking due to differences in study population characteristics, heterogeneity in study designs, and discordance in defining insomnia [5]. Nonetheless, the worldwide prevalence rate of co-occurrence of insomnia with OSA ranges between 18% and 42% while the prevalence rate in those patients presenting for treatment is estimated at 29–67% [6]. 

Numerous epidemiologic and cohort-based studies have documented that COMISA imparts a worse quality of life, greater daytime impairments, reduced productivity, and more serious mental ailments in comparison to individuals with either OSA or insomnia alone [7–10]. Despite the established benefits of available therapeutic modalities for each sleep disorder, there is no consensus or approved guideline on the best approach or sequence of treatment. Continuous positive airway pressure (CPAP) is the most effective therapy for OSA [11] while cognitive behavioral therapy for insomnia (CBT-I) is deemed the gold standard treatment for insomnia [12]. The traditional approach to the management of COMISA is to institute therapy for each disorder independently. However, clinical evidence suggests that the clinical benefits from such a regimen are not always guaranteed or predictable. Patients with COMISA are faced with several challenges. First, the presence of comorbid insomnia reduces CPAP adherence [10, 13, 14]. The prolonged sleep onset latency heightens an individual’s sensitivity to pressurized CPAP masks making use of CPAP less desirable. Second, access to trained behavioral specialists represents a barrier to patients seeking CBT-I, particularly in rural areas [15]. Third, pharmacologic treatment of insomnia without adequate management of OSA poses a significant risk of worsening daytime sleepiness and cognitive impairment.

Examining the clinical characteristics of patients with COMISA who respond to CBT-I would assist in identifying phenotypic traits that would contribute to expedited delivery of care and lead to improved outcomes. To achieve these goals, we used data from the US Veterans Health Administration (VHA) services to delineate clinical features predictive of a favorable insomnia response following Brief Behavioral Therapy for Insomnia (BBTI)-an adapted version of CBT-I.

## Methods

### Study population

Regular VHA users, 18 years of age and older, diagnosed with obstructive sleep apnea between April 2018 and June 2024 based on the International Classification of Diseases-Tenth Revision (ICD-10) (G47.33) at the VA Western New York Healthcare System were identified through the VHA Corporate Data Warehouse. Regular use of VHA was defined as at least 2 years of healthcare utilization either as an encounter or prescription refill prior to entry into the cohort. Veterans who only had visits for prosthetics or hearing aids were excluded. The diagnosis of OSA was confirmed when the ICD-10 codes were listed on two separate occasions more than 30 days apart but within 390 days of each other [16]. In this patient group, cases of newly diagnosed insomnia between January 2021 and December 2023 corresponding to established ICD-10 codes (F51.01, F51.03, F51.04, F51.05, F51.09; G47.00, G47.01, and G47.09) were selected. The date of insomnia diagnosis was considered the index date. Given that the diagnosis of sleep apnea had to precede the index date, the cohort of COMISA patients comprised cases between January 2021 and December 2023. Exclusion criteria included the diagnosis of central apnea (G47.37), restless leg syndrome (G25.81), parasomnias (G47.5), circadian rhythm disorders (G47.20), and alcohol and substance use disorders (F10.2, F11.20, F19.9). Eligible cases were reviewed manually by accessing the computerized patient record system. Individuals who received hypnotics/sedatives (i.e. benzodiazepines, Z-drugs, dual orexin receptor antagonists, doxepin, trazodone) in the 3 months prior to the index date for insomnia were excluded. Patients receiving opioids or using devices other than CPAP for OSA were also not included. Cases with unstable medical or mental conditions defined as hospitalization, emergency room visit, or a change in medication use within 3 months were not considered in the analysis. The study was approved by the VA Research and Development Committee. The requirement for written informed consent was waived because the study met the exemption criteria from the Common Rule. The study was conducted in accordance with the guidelines of Strengthening the Reporting of Observational Studies in Epidemiology [17]. 

### Covariates

Collected variables comprised patients’ demographic and anthropomorphic data, medications, and relevant comorbid conditions. The propensity for sleepiness was assessed by the Epworth Sleepiness Scale (ESS) [18]. A score of 10 or higher is considered the cutoff point for indicating excessive daytime sleepiness. Polysomnographic data at the time of OSA diagnosis was retrieved. Apneas were defined as a ≥ 90% reduction in respiratory amplitude for ≥ 10 s. Hypopneas were defined as a ≥ 30% reduction in respiratory amplitude for ≥ 10 s associated with a ≥ 3% oxygen desaturation or an arousal [19]. Severity of OSA was obtained from the apnea-hypopnea index (AHI) which corresponded to the total number of apneas and hypopneas calculated per hour and was graded as mild for AHI ≥ 5 and < 15, moderate for AHI ≥ 15 and < 30, and severe for AHI ≥ 30.

### Intervention

The version of CBT-I administered at our institution is the Brief Behavioral Therapy for Insomnia (BBTI), an evidence-based adapted version of CBT-I that combines ease of administration with a flexible treatment delivery [20]. In brief, BBTI was delivered in an individual format over six consecutive weeks by a trained behavioral sleep provider and included one individual in-person visit (45 min) at week 1, a second in-person visit at week 3 (< 30 min), and brief telephone appointments during weeks 2 and 4 (< 20 min each). The first session is designed to be both educational and interactive. A recommended bedtime and rise time were determined based on data gathered from the baseline sleep diary. A customized sleep schedule was formulated to limit bed rumination. A minimum bedtime was set at 6 h for safety reasons even if the patient reported sleeping fewer than 6 h (sleep restriction). In addition, patients received instructions on how to avoid going to bed unless sleepiness had set in and to get out of bed if awake for more than 30 min (stimulus control). The second in-person visit was dedicated to reinforcing adherence to the prescribed sleep-wake schedule and determining whether changes in the prescribed time in bed are required. The two phone contacts were designed to troubleshoot any emerging issues regarding adherence to the sleep schedule. The sessions were used also to review the rules for better sleep, including the instructions for stimulus control and sleep restriction.

### Measurements

The Epworth Sleepiness Scale (ESS) [18] and the Insomnia Severity Index (ISI) [21] were extracted at baseline and 12±1 weeks after BBTI therapy. The ESS, which is a self-administered questionnaire, rates subjects on a scale of 0 to 3 based on the potential of falling asleep in eight different situations. The final score ranges from 0 (no daytime sleepiness) to 24 (maximum daytime sleepiness). ESS has a high level of internal consistency (α = 0.88) and a high test-retest reliability [22].

The ISI is a 7-item patient-reported outcome assessing the severity of initial, middle, and late insomnia; sleep satisfaction; interference of insomnia with daytime functioning; noticeability of sleep problems by others; and distress about sleep difficulties. A 5-point scale is used to rate each item, yielding a total score ranging from 0 to 28. A higher score indicates more severe insomnia within the following 4 severity categories: absence of insomnia (score of 0–7); subthreshold insomnia (score of 8–14); moderate insomnia (score of 15–21); and severe insomnia (score of 22–28). The ISI has excellent internal consistency (Cronbach α = 0.74) and temporal stability (*r* = 0.80) [21]. A positive response to BBTI was defined as a reduction in ISI score of ≥ 8 from baseline. Remission from insomnia referred to a post-treatment ISI score of < 8 [23]. Those who were prescribed hypnotics or sedatives between the end of BBTI sessions and the subsequent measurement of ISI were assigned their corresponding baseline ISI score.

### CPAP usage

At the time of OSA diagnosis, all patients received a standardized education session with their bed partner (if possible) designed to improve their CPAP adherence. A respiratory therapist provided an educational brochure that detailed the regular cleaning of CPAP accessories including how to troubleshoot mask-related problems. All CPAP units were equipped with built-in modems designed to upload daily utilization data to a cloud-based platform (Encore Pro Software; Respironics Inc. Murrysville, PA). CPAP utilization was calculated based on average hours of use per night. CPAP adherence was defined as use of PAP ≥ 4 h per night on 70% of nights during a consecutive thirty (30) day period.

### Statistical analysis

Statistical analyses were performed with STATA software, version 16 (StataCorp, College Station, TX). Descriptive statistics were presented as mean values (with SD) for continuous variables and as frequencies (with percentages) for categorical variables. The Shapiro-Wilk test was used to assess the normality of continuous variables. Differences between BBTI responders and BBTI nonresponders were tested with the Chi-square test or the Fisher exact test for categorical variables and with the *t* test or Wilcoxon rank sum test for continuous variables. To determine predictive factors associated with favorable response to BBTI, we run generalized linear model analyses after controlling for potential confounding covariates in our conceptual framework. Covariates with *p* values of ≤ 0.20 in our univariate analysis were entered into the multivariate model. Selected variables were checked for multicollinearity by correlation matrix and interactions terms. The odds ratio (OR) and the 95% confidence intervals (CI) for all independent variables were reported. A receiver operating characteristic (ROC) curve was constructed to evaluate the accuracy of selected covariates for predicting BBTI responders. The diagnostic value is considered acceptable when the area under the ROC curve (AUC) is between 0.71 and 0.8, excellent when it is 0.81–0.9, and outstanding when it is > 0.9 [24]. Youden’s index (sensitivity + specificity − 1) was used to select the most suitable cut-off values for predicting BBTI responders [25]. Missing values at follow-up were assumed to be missing at random and were imputed using a regression-switching approach (chained equations with *m* = 5 imputations) using all patient characteristics with a predictive mean‐matching method for quantitative variables and a logistic regression model for categorical variables. A statistically significant difference was considered if *P* value < 0.05.

## Results

**Fig. 1:**
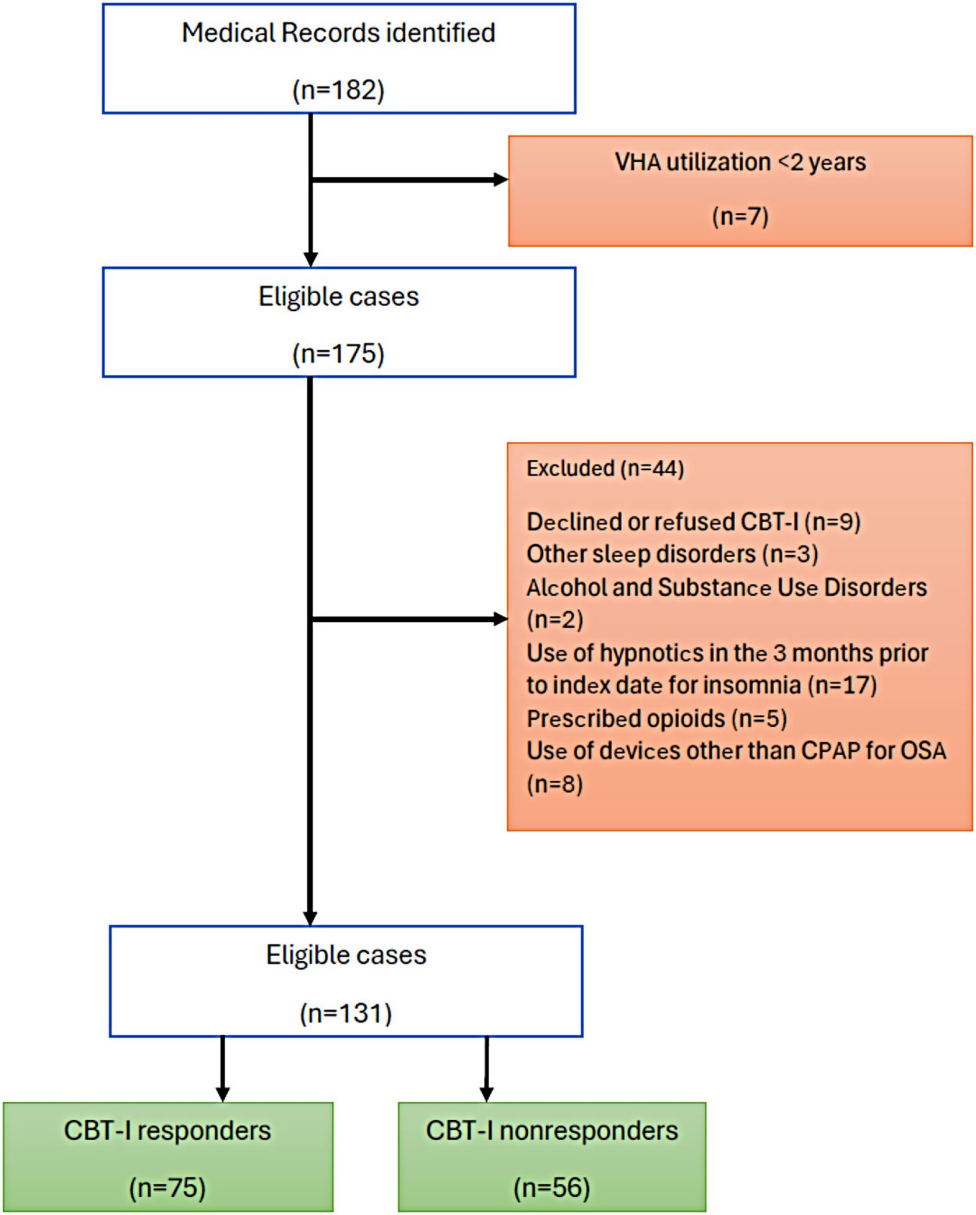
Flow diagram depicting the selection of the study population

Querying the CDW identified 182 veterans with ICD-10 diagnostic codes corresponding to coexistent OSA and insomnia who were referred to BBTI between January 2021 and December 2023. Figure 1 depicts the selection of the study cohort. Seven cases were excluded for administrative purposes. An additional 44 patients met the exclusion criteria, leaving 131 for statistical analysis. The mean age of the study cohort was 48.5±9.2 years with 85% being male and 69% Caucasians. Most patients (69%) were married or living with someone with 54% employed, and 19% retired. The average BMI was 27.4 ± 3.9 kg/m^2^ with obese patients (BMI ≥ 30 kg/m^2^) accounting for 26% of the total study population. A history of active smoking was present in 25% of the study cohort. Coronary artery disease (35%), chronic obstructive pulmonary disease (18%), and diabetes mellitus (11%) were the predominant medical comorbidities while posttraumatic stress disorder (PTSD) and depression represented 68% and 34% of all psychiatric comorbidities, respectively.

Sleep apnea severity was categorized as mild in 31%, moderate in 37%, and severe in 31%. All patients underwent initially an in-laboratory CPAP titration following OSA diagnosis. The mean CPAP pressure for the cohort was 9.2 ± 3.4 cm H_2_O. Most patients were being treated with a nasal pillow as the most common interface (63%) followed by a full facemask (22%) and nasal mask (15%). In retrospect, insomnia symptoms were present 9.7 ± 2.3 years prior to the diagnosis. Among the 131 patients with COMISA, 44% had sleep onset insomnia, 46% had sleep maintenance, and 32% had early awakening, with 23% having more than one type of insomnia. The average ISI for the cohort was 18.6 ± 3.9 indicating moderate to severe insomnia.

### Response to BBTI

Patients completed on average 3.35 ± 1.58 sessions for BBTI. Thirty-eight patients (29%) missed at least one BBTI session, of whom 22 dropped out prior to the fourth session. Comparisons between the 38 patients who did not complete treatment and the 93 patients who did revealed no significant difference with respect to age, sex, prevalence of medical or psychiatric disorders, or marital status. However, a higher dropout rate was observed among nonwhites (*p* = 0.02). Following BBTI treatment, the ESS dropped from 12.3 ± 4.6 to 10.4 ± 4.1 (difference − 1.8; 95% CI [-2.5, -1.1]; *p* < 0.001) and ISI from 18.6 ± 3.9 to 10.7 ± 5.4 (difference − 7.9; 95% CI [-8.8, -6.9]; *p* < 0.001). Fifty-six patients (43%) did not meet ISI criteria for BBTI response.

Pretreatment demographic characteristics and clinical variables by BBTI response are summarized in Table 1. No significant differences in age, sex, BMI, or marital status were observed between BBTI responders and BBTI nonresponders. Similarly, neither type of insomnia nor the presence of underlying comorbidities was considerably different between the two groups. However, the prevalence of Caucasians was significantly higher in BBTI responders compared with BBTI nonresponders (*p* = 0.006). The duration of insomnia symptoms prior to diagnosis tended also to be shorter in BBTI responders compared with BBTI nonresponders although the difference did not attain statistical significance (*p* = 0.05).

**Table 1:** Baseline study population characteristics

	BBTI responders (*n* = 75)	BBTI nonresponders (*n* = 56)	*P* value
Age, years	48.6 ± 8.7	48.5 ± 10.1	0.96
Sex, (M/F)	60/15	48/8	0.39
**Race**			0.006
Caucasians	60 (80)	31 (55)	
African Americans	13 (17)	24 (43)	
Others	2 (3)	1 (2)	
**BMI**, kg/m^2^	27.5 ± 3.1	27.4 ± 4.9	0.83
**Marital status**, n (%)			0.42
Married	50 (67)	41 (73)	
Other	25 (33)	15 (27)	
**Smoking history**, n (%)			0.62
Active smokers	23 (31)	15 (27)	
Past or never smokers	52 (69)	41 (73)	
**Insomnia duration, years**	9.4 ± 2.3	10.1 ± 2.4	0.05
**Type of Insomnia**, n (%)			
sleep onset	30 (40)	28 (50)	0.25
maintenance	37 (49)	23 (41)	0.35
Early morning	25 (33)	17 (30)	0.72
**AHI**, h^-1^	23.5 ± 11.6	27.3 ± 20.2	0.21
Medical comorbidities, n (%)	38 (51)	24 (43)	0.38
Psychiatric comorbidities, n (%)	46 (61)	31 (55)	0.49
**Medications, n (%)**			
Antidepressants	16 (21)	10 (18)	0.62
Antipsychotics	2 (3)	3 (5)	0.73

AHI = Apnea Hypopnea Index; BBTI = Brief Behavioral Treatment for Insomnia; BMI = Body Mass Index

Table 2 summarizes the qualitative and quantitative sleep assessments in the study population. Patients who did not respond to BBTI had a higher sleep propensity at baseline than those who did respond to BBTI (*p* = 0.02), however, both groups had comparable degree of insomnia severity (*p* = 0.78). A comparison of polysomnographic parameters at baseline revealed no significant difference in SOL, sleep stages, or arousal index between BBTI responders and BBTI nonresponders. Only total sleep time was significantly shorter in BBTI nonresponders compared to BBTI responders (difference − 76.23 min, 95% CI [-105.04, -47.42]; *p* < 0.001).

**Table 2:** Qualitative and quantitative sleep assessments

	BBTI responders (*n* = 75)	BBTI nonresponders (*n* = 56)	*P* value
**Sleep surveys**			
ESS	11.5 ± 4.6	13.1 ± 3.9	0.02
ISI	18.7 ± 4.3	18.5 ± 3.6	0.78
**Baseline PSG**			
SOL, min	35.5 ± 28.5	40.7 ± 27.4	0.29
TST, min	319.6 ± 71.3	243.3 ± 97.6	< 0.001
N1, %	7.2 ± 5.1	7.9 ± 4.5	0.52
N2, %	67.9 ± 11.9	66.7 ± 17.6	0.65
N3, %	11.2 ± 7.4	12.8 ± 9.4	0.21
REM, %	15.6 ± 8.2	15.1 ± 11.2	0.75
**SpO**_2_ **, nadir %**	85.3 ± 2.4	84.7 ± 4.6	0.36
Arousal Index, h^-1^	8.8 ± 11.6	9.4 ± 7.7	0.65

BBTI = Brief Behavioral Treatment for Insomnia; ESS = Epworth Sleepiness Scale; ISI = Insomnia Severity Index; TST = Total Sleep Time; PSG = Polysomnogram; REM = Rapid Eye Movement

### CPAP response to BBTI

The mean CPAP pressure for the BBTI responders was 9.1 ± 4.1 cm H_2_O and 9.5 ± 3.6 cm H_2_O for BBTI nonresponders, respectively (*p* = 0.52). At baseline, the mean CPAP usage across all nights was 82.2 ± 64.8 min. Prior to BBTI, there was no significant difference in the CPAP usage between BBTI responders (84.7 ± 53.9 min) and BBTI nonresponders (78.9 ± 77.5 min) (difference 5.7 min, 95% CI [-18.3, 29.7]; *p* = 0.62). Post BBTI, patients in BBTI responders used CPAP for a longer duration compared with baseline (difference 63.6 min, 95% CI [51.1, 76.3]; *p* < 0.001] but not for BBTI nonresponders (difference 9.1 min, 95% CI [-10.2, 28.4]; *p* = 0.35). Moreover, BBTI responders achieved higher CPAP use (148.3 ± 84.4 min) than BBTI nonresponders (84.6±53.9 min) (difference 60.3 min, 95%CI [30.3, 90.2]; *p* < 0.001)). CPAP adherence was also higher in BBTI responders than nonresponders, but the difference was not statistically significant between the two groups (21% versus 12%, respectively; *p* = 0.29). The incidence of adverse effects related to the mask (air leak, sinus pressure, claustrophobia, and bloating) was not significantly different between BBTI responders and BBTI nonresponders. Lack of adherence was attributed to sleep disturbances (42%) and difficulty in falling asleep while wearing the mask (33%).

**Fig. 2:**
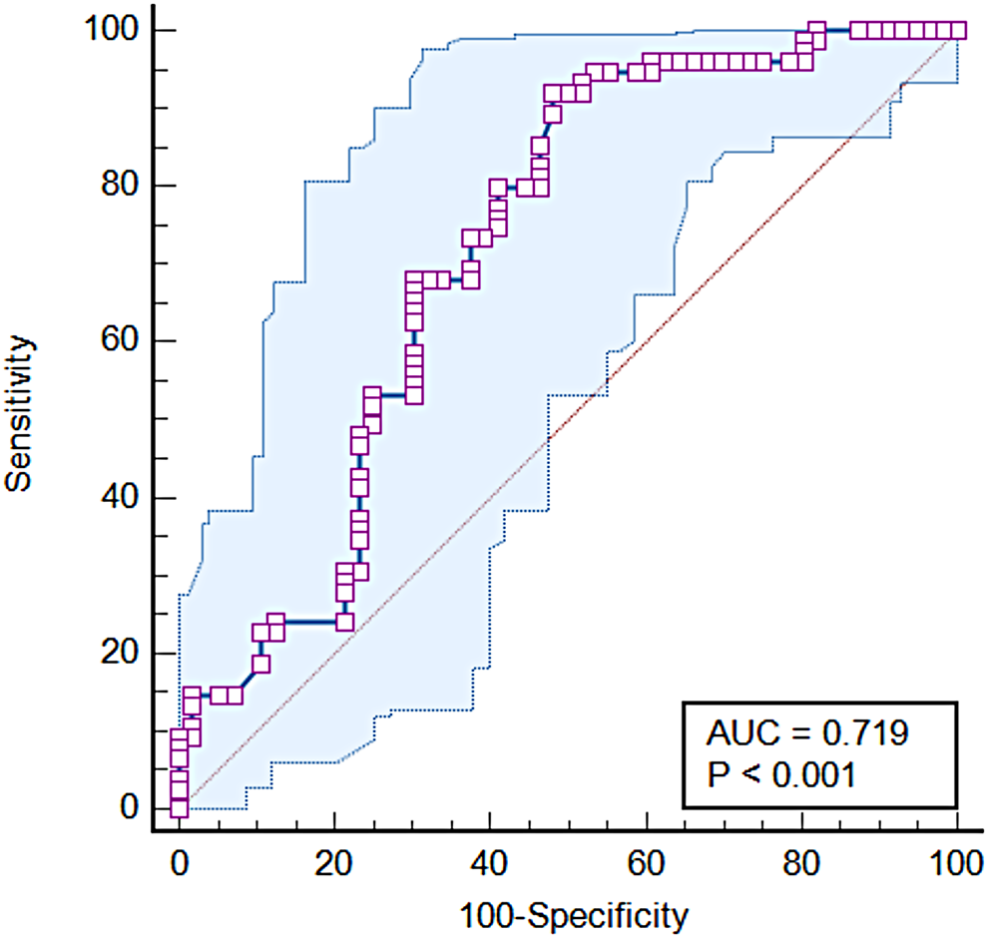
A receiver operator characteristic curve for total sleep time. AUC = area under the curve; TST = total sleep time

### Predictors of BBTI response

**Fig. 3:**
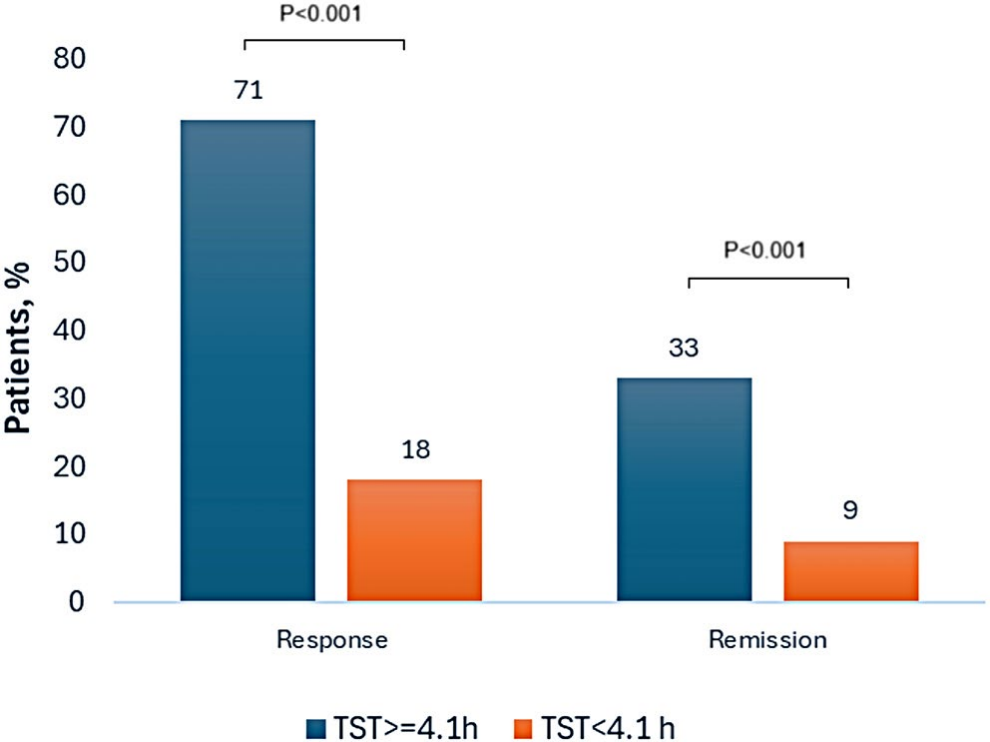
Response and remission rates based on optimal total sleep time

To identify factors predictive of a positive response to BBTI, the binary dependent variable was regressed onto the independent variables of race, ESS, insomnia duration, and PSG-derived TST (model1) (Table 3). Non-whites (OR 3.5, 95% CI [1.4, 8.8]) and shorter sleep time (OR 0.98, 95% CI [0.98, 0.99] were independent predictors of poor insomnia response to BBTI. These findings remained true even with forcing depression and AHI into the regression model (model 2). The AUC for TST was 0.72 (95%CI 0.63–0.81) with an optimal cut-point of < 4.1 h (χ^2^ = 29.4, *p* < 0.001) (Fig. 2). With this threshold, 71% of patients with TST ≥ 4.1 h had responded favorably to BBTI compared to only 18% with TST < 4.1 h while remission was achieved in 33% and 9%, respectively (*p* < 0.001) (Fig. 3). This indicates that TST duration ≥ 4.1 h is a reliable indicator for the predictive values of response outcome to BBTI.

**Table 3:** A multivariate analysis identifying factors associated with favorable response to BBTI

	OR (95% CI)	Std Error	*P* value
Model 1			
Race	0.28 (0.12, 0.69)	0.13	0.006
ESS	0.95 (0.85–1.07)	0.06	0.47
Insomnia Duration	0.85 (0.66, 1.04)	0.09	0.12
TST*	1.01 (1.006, 1.02)	0.003	< 0.001
Model 2			
Depression	1.74 (0.18, 35.56)	0.78	0.22
AHI	0.99 (0.97, 1.02)	0.01	0.79
Race	0.28 (0.11, 0.72)	0.14	0.008
ESS	0.94 (0.86, 1.07)	0.06	0.35
Insomnia Duration	0.85 (0.62, 1.004)	0.09	0.14
TST*	1.01 (1.006, 1.02)	0.003	< 0.001

OR = Odds Ratio; ESS = Epworth Sleepiness Scale; AHI = Apnea Hypopnea Index; TST*=Total Sleep Time derived from polysomnography

## Discussion

Although clinical trials have demonstrated the clinical effectiveness of cognitive behavioral therapy for insomnia in patients with COMISA [26–28], identifying uniform clinical determinants of favorable response to cognitive behavioral therapy in this population have been elusive. While the results of this study reiterate the benefits of BBTI in reducing the severity of insomnia and improving CPAP utilization, it highlights the heterogeneity of insomnia coexisting with OSA by recognizing race and short sleep duration to be significant predictor variables of BBTI outcomes. More importantly, it introduces a novel conceptual framework that goes beyond the traditional approach of one-size-fits-all. It underscores the relevance of distinct sociodemographic and polysomnographic traits that could be incorporated into future treatment strategies of patients with COMISA.

The effect of race on cognitive behavioral therapy for insomnia on outcomes has been an area of increasing focus in recent years [29, 30]. Racial and ethnic disparities in sleep disorders, notably insomnia, are well-documented, and these disparities can influence the effectiveness of psychological interventions, including BBTI. Cognitive behavioral therapy was originally developed and tested primarily within white populations, which may not align with the values, or lived experiences of non-white individuals. For example, cognitive behavioral therapy emphasizes changing individual thoughts and behaviors, whereas many non-Western cultures strongly emphasize community, family, and collective identity. This cultural mismatch can make it difficult for some individuals to relate to, or fully engage with, the approach that CBT uses. Unfortunately, cultural adaptation of BBTI across various racial and ethnic groups is still a challenge [31]. A recent meta-analysis of RCTs conducted in the United States that incorporated behavioral interventions for the management of sleep-wake disorders found that less than 7% of the published literature targeted racial/ethnic minorities, immigrants, or sexual orientation minorities [31]. Other systematic reviews did not even evaluate the impact of race and ethnicity on intervention efficacy [32, 33]. The American Academy of Sleep Medicine acknowledged this information gap in its most recent guideline on behavioral and psychological treatments of chronic insomnia disorder [34]. The current study provides additional insights into the reasons for the reduced effectiveness of BBTI in non-whites. First, although the frequency of non-completers was in line with other studies that reported 14–40% failure to complete the full course of CBT-I [35, 36], non-whites represented a larger share of those who dropped out from CBT-I. It was unclear from the records whether withdrawing from BBTI therapy was either because participants were unsatisfied with treatment, developed complications, lost interest, or simply were unable to meet the time commitment. Future research ought to incorporate patient expectations, patient satisfaction, and barriers to accessing mental health care, including affordability issues, and past negative healthcare experiences. Second, BBTI is typically not tailored to address culturally specific stressors or sleep disturbances unique to certain communities, such as discrimination or socio-environmental stress [31]. Such unaddressed issues can impact the effectiveness of BBTI for non-white individuals, as their sleep challenges may stem from systemic stressors not addressed in traditional CBT-I frameworks [37]. Third, in many non-white communities, mental health issues are often stigmatized, and seeking therapy can be seen as a sign of weakness or failure [38]. This stigma can reduce engagement, and individuals may be less likely to fully participate in BBTI exercises. Fourth, cultural norms around sleep and family obligations may differ across racial and ethnic groups [39, 40]. For instance, multi-generational households or late-night family activities are more common in some cultures, and this can affect sleep routines and adherence to BBTI recommendations on sleep hygiene. If BBTI fails to account for these lifestyle differences, its applicability may be limited.

In terms of clinical implications, the current study emphasizes considering the duration of sleep as a modifiable factor likely to enhance response to BBTI. Specifically, analysis of the existing data revealed that patients who had a TST < 4.1 h at baseline were less likely to experience significant relief from insomnia after BBTI than those with TST ≥ 4.1 h. Given that the core of BBTI entails consolidating fragmented sleep through controlled sleep deprivation, imposing further restrictions on restorative sleep in these patients may not improve total sleep time, limiting the effectiveness of the treatment. Furthermore, it has been shown that patients within insomnia of short sleep (ISS) duration have higher baseline levels of physiological arousal, a state often characterized by increased cortisol, heart rate, and sympathetic nervous system activity [41, 42]. This hyperarousal—both mental and physiological—confers a “BBTI resistant” status, for BBTI primarily targets cognitive and behavioral factors rather than directly addressing heightened physiological arousal. Other studies that have supported the TST binary categorization have used a threshold of less than 6 h [43, 44]. This 6-hour cutoff was arbitrarily selected based on the risk of incident hypertension [45]. In contrast, two previous studies have identified a cut-point of TST < 3.65 h and TST < 4.1 h for predicting dropout from CBT-I [46, 47]. Based on the current study, the TST threshold for identifying BBTI-nonresponders appears to substantiate a much shorter sleep duration than what currently defines ISS. The fact that the response to BBTI in ISS patients with TST < 6 h has been largely divergent indicates a suboptimal cutoff [43, 48, 49]. While it can be argued that adherence to BBTI is not synonymous with suboptimal response, the outcome in terms of remission from insomnia is not much different [50]. Testing a lower TST threshold could improve the overall treatment of insomnia by providing clinicians with better guidance to match treatment strategies with patient characteristics.

Not surprisingly, a poor response to BBTI in COMISA patients can have a profound negative impact on CPAP adherence due to persistent insomnia, negative perceptions of CPAP, and ongoing sleep fragmentation. For these patients, the persistence of insomnia symptoms despite BBTI can reinforce negative associations with sleep and heighten pre-sleep arousal, making it difficult to tolerate CPAP use. Many individuals with COMISA already struggle with fragmented sleep, and when insomnia remains unresolved, the discomfort or inconvenience of CPAP therapy may become an additional perceived barrier to restful sleep. A critical factor in this dynamic is the overlap between reported sleepiness and perceived insomnia. Patients with COMISA may attribute their daytime fatigue primarily to poor sleep quality from insomnia rather than to the undertreated sleep apnea, leading them to prioritize insomnia-focused interventions while neglecting CPAP therapy. Others may mistake residual sleepiness from suboptimal CPAP adherence as a sign that BBTI treatment is ineffective, further discouraging its consistent use. Ultimately, in patients with COMISA who exhibit a poor response to BBTI, a more integrative approach may be necessary to improve CPAP adherence.

Several limitations of this investigation should be noted. First, retrospective analyses are subject to hidden biases and unmeasured confounding. More importantly, we have relied on billing codes to develop our database. As with any large observational study using administrative claims data, the risk of inaccurate or missing covariates should be taken into consideration. Second, these results are based on a sample of patients consisting exclusively of veterans. Although other studies with more diverse populations have reached similar conclusions, OSA in the veteran population presents distinct pathophysiologic characteristics influenced by high rates of medical or behavioral comorbidities, environmental exposures, and service-related factors. Unlike the general population, veterans exhibit a higher prevalence of the low arousal threshold endotype of OSA which involves repeated arousals triggered by minimal breathing effort, prematurely interrupting the buildup of ventilatory drive. Since this increased central respiratory drive is essential for activating upper airway dilator muscles, early arousals interfere with the progression into deeper slow-wave sleep and disrupt breathing stability—both crucial for maintaining pharyngeal patency [51]. The presence of coexisting insomnia in concert with PTSD further exacerbates this hyperarousal state, creating a self-reinforcing cycle that amplifies respiratory instability [52]. Third, our findings pertain specifically to BBTI. When comparing CBT-I to BBTI, clinical investigations have generally shown that both treatments can reduce insomnia severity, but CBT-I may demonstrate slightly better outcomes due to its more comprehensive approach, including cognitive restructuring, while BBTI is designed to be shorter, more accessible and delivered with fewer sessions, focusing primarily on behavioral components like sleep restriction and stimulus control [53]. Fourth, the outcomes of the study were based on self-report data. While this is true, the selected outcomes are subjective by their very nature. In addition, all variables were based on standardized questionnaires. Nevertheless, future trials should include objective measures of the BBTI success, including polysomnographic sleep parameters. Fifth, although we excluded cases in which medication changes occurred within the three months preceding insomnia diagnosis, the interaction between routinely prescribed psychotropic medications and sleep could still have impacted the study’s outcome.

In conclusion, we found that nonwhites and shorter sleep duration were associated with poor response to BBTI. These results suggest that specific insomnia phenotypic traits should be considered in the treatment of patients with COMISA.

## Data Availability

Data will be made available on request to the corresponding author on reasonable request.

## Declarations

### Disclaimer

The views expressed in this article are solely those of the authors and do not reflect an endorsement by or the official policy of the U.S. Army, the Department of Defense (DoD), the Department of Veterans Affairs (VA), or the U.S. Government. This material is the result of work supported with resources and the use of facilities at the VA Western New York Health Care System.
